# Exploring the potential of late permian aggregate resources for utilization in engineering structures through geotechnical, geochemical and petrographic analyses

**DOI:** 10.1038/s41598-023-32294-0

**Published:** 2023-03-29

**Authors:** Javid Hussain, Jiaming Zhang, Syed Muhammad Iqbal, Jabir Hussain, Fitriani Fitria, Xiao Lina, Nafees Ali, Sartaj Hussain, Waseem Akram, Mubasir Ali

**Affiliations:** 1 Department of Geological Engineering, China University of Geosciences (Wuhan), Wuhan, 430074 China; 2 State Key Laboratory of Geo-Mechanics and Geotechnical Engineering, Institute of Rock and Soil Mechanics, Chinese Academy of Sciences, Wuhan, China; 3 Department of Earth & Environmental Sciences, Bahria University, Islamabad, Pakistan; 4 Department of Geophysics and Geomatics, China University of Geosciences (Wuhan), Wuhan, 430074 China; 5 Zijin Mining Group Company Limited, Shanghang, 364200 Fujian China; 6 Department of Earth Resources, China University of Geosciences (Wuhan), Wuhan, 430074 China

**Keywords:** Civil engineering, Geology

## Abstract

The China-Pakistan Economic Corridor (CPEC) is an ongoing mega-construction project in Pakistan that necessitates further exploration of new natural resources of aggregate to facilitate the extensive construction. Therefore, the Late Permian strata of Chhidru and Wargal Limestone for aggregates resources were envisaged to evaluate their optimal way of construction usage through detailed geotechnical, geochemical, and petrographic analyses. Geotechnical analysis was performed under BS and ASTM standards with the help of employing different laboratory tests. A simple regression analysis was employed to ascertain mutual correlations between physical parameters. Based on the petrographic analysis, the Wargal Limestone is classified into mudstones and wackestone, and Chhidru Formation is categorized into wackestone and floatstone microfacies, both containing primary constituents of calcite and bioclasts. The geochemical analysis revealed that the Wargal Limestone and Chhidru Formation encompass calcium oxide (CaO) as the dominant mineral content. These analyses also depicted that the Wargal Limestone aggregates bear no vulnerability to alkali-aggregate reactions (AAR), whereas the Chhidru Formation tends to be susceptible to AAR and deleterious. Moreover, the coefficient of determination and strength characteristics, for instance, unconfined compressive strength and point load test were found inversely associated with bioclast concentrations and directly linked to calcite contents. Based on the geotechnical, petrographic, and geochemical analyses, the Wargal Limestone proved to be a significant potential source for both small and large-scale construction projects, such as CPEC, but the Chhidru Formation aggregates should be used with extra caution due to high silica content.

## Introduction

Due to the high demand for concrete, a significant volume of natural resources is required^1^, and modern construction entails concrete as a fundamental element which is a blend of fine to coarse aggregates, water, and cement that can be molded before setting up into a tight and solid mass^2^. Many civil engineering projects use aggregates as reinforcement, as well as to decrease shrinkage and to provide economic benefit^3^. According to Kim^4^, in concrete, aggregates compose 75 to 85% of the mixture, while asphalt mixtures make up 93 to 95%, and rail ballast and road base constitute nearly 100% of the mixture. Therefore, it is imperative to examine the chemical, physical, mechanical, and mineralogical properties of the aggregates due to their effects on the strength and durability of the concrete^5,6^, other than the extensive use in construction. As a geotechnical material and an aggregate of crushed rock, limestone plays a major role in the construction industry based on its physical and mechanical properties^7^. The physicomechanical and durability qualities of the crushed rock aggregates are greatly influenced by the petrographic features of subsequent processes and source rock, such as faulting, weathering, folding, and hydrothermal activity^8^. These physicomechanical and petrographic characteristics may be affected by the mineral contents, hardness, chemical stability, porosity, and composition. It is important to analyze the petrography of aggregates in order to identify its texture, mineralogy, bioclasts, matrix type, microfractures, and texture type^9^. Some scholars have examined and made predictions about the engineering qualities of aggregates based on their petrographical and physical characteristics^6,10^.

The geotechnical and rock engineering fields use various rock classification systems which are primarily based on mechanical parameters such as uniaxial compressive strength, Young's modulus, tensile strength, Poisson's ratio, and point load tests. Nonetheless, It is the mineral composition of a rock that determines whether it is suitable or not for use as a construction material^1^. The impact of physicomechanical qualities on aggregate quality is of paramount significance, other than the concrete-related features, including alkali-aggregate reaction (AAR), durability, and strength must also be taken into consideration^11^. The strength, performance, and durability of concrete may be compromised if adequate measures are not taken to prevent AAR^12^. In the presence of certain reactive minerals, like strained SiO_2_ and CaMg(CO_3_)_2_, alkalis react to produce Alaklai silica reaction (ASR) and Alakali carbonate reaction (ACR), respectively^8,13^. After years of research, subsequently, it was shown that certain aggregates are not only reactive but also produce strong linkages at the periphery levels of both the aggregate and the mixture. Therefore, Using petrographic and chemical analyses in structural concrete, reactive and non-reactive minerals can be detected, reaction rims, silicate gels, micro/macro/macro structural properties, and carbonation can be assessed^13^.

**Figure 1:**
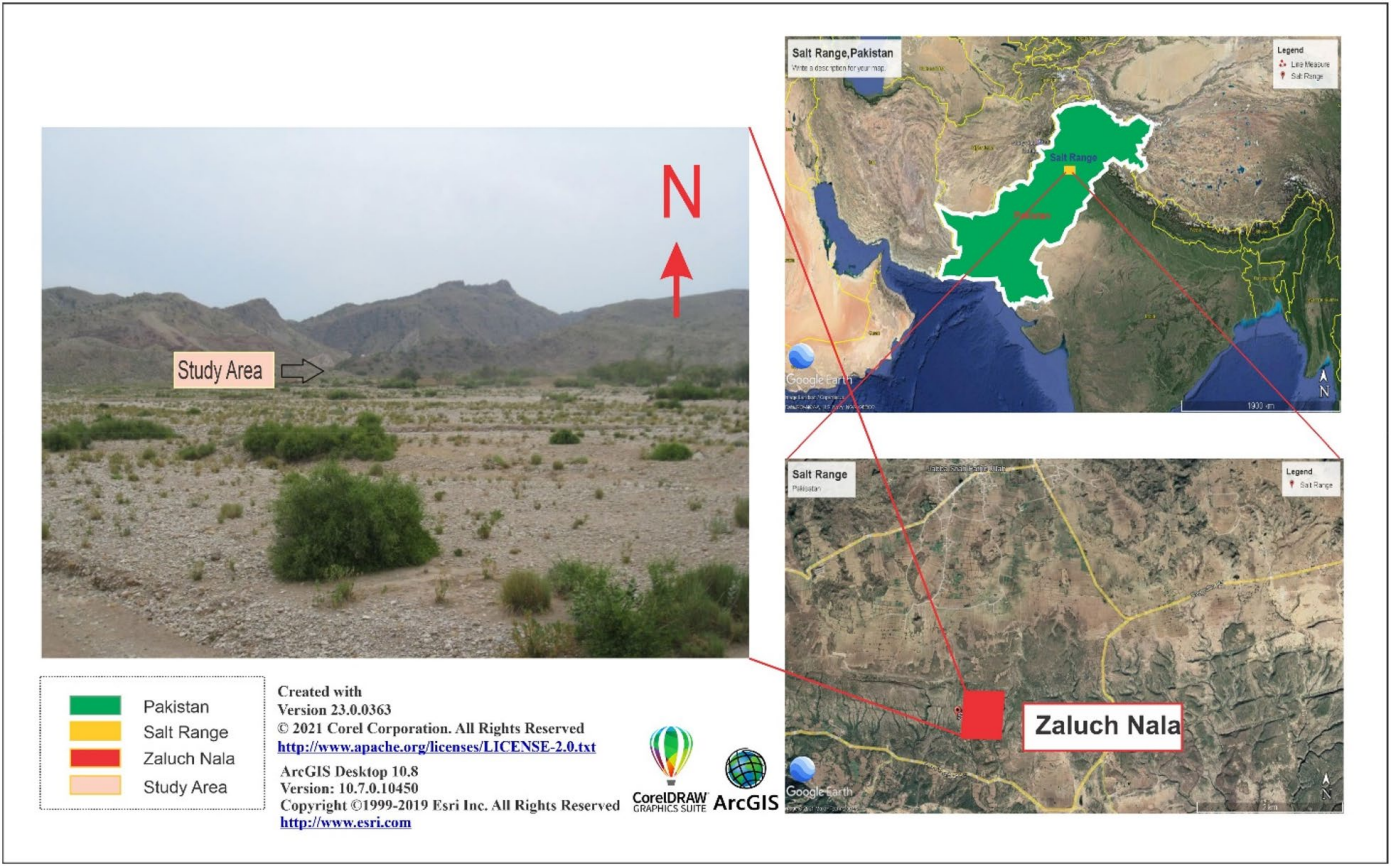
Location map of the study area, Salt Range, Pakistan.

Major sources of aggregate in Pakistan include limestones obtained from Margala hills, aggregate from the Kirana hills, District Khyber hills, and carbonates of Wargal, Sakasar, and Kohat^12,14^. Mostly Palaeocene and Eocene sedimentary successions of Pakistan have the utmost significance in terms of geotechnical use and hydrocarbon exploration^15,16^. The National Highway Authority (NHA) exploited these aggregates to build roadways as part of the massive China-Pakistan Economic Corridor (CPEC) project. The CPEC project connected over 70 countries via the Gwadar port of Pakistan^17^. The CPEC includes several projects of both short and long terms, such as building roads, railroads, and fiber optics, however, with the sharp rise in demand for construction materials brought on by the nation's rapid population expansion and development of small and mega-projects, current aggregate resources are running short. Therefore, many exploratory research studies for prospects, such as aggregate for the development of construction resources are required to satisfy the future demands of the construction activities significantly^11^. Researchers in Pakistan have conducted several studies on aggregate and limestone to study their mechanical properties, make engineering assessments and aggregate assessments, and proposed their use in construction. (e.g. Naeem et al.^5^; Naseem et al.^18^; Majeed and Abu Bakar^19^; Mustafa et al.^20^; Akram et al.^20^; Rehman et al.^21^; Ullah et al.^22^, Kamran et al.^23^; Zada et al.^16^. Physicochemical, geochemical, and petrographic analyses play a crucial role in determining the suitability of aggregates for construction. Keeping the significance of the region in mind, the present research employs these analyses to investigate the Wargal Limestone and Chhidru Formation exposed in the Zaluch Group, Western Salt Range (Fig. 1) in an in-depth and comprehensive manner to evaluate the feasibility of these aggregate resources for CPEC and Pakistan's current massive-scale engineering development projects. Furthermore, the findings of this research would offer recommendations and instructions for comprehending the mechanical behavior of rock units and their subsequent excavation and use in local geotechnical and construction sectors.

### Geology and stratigraphy of the area

**Figure 2:**
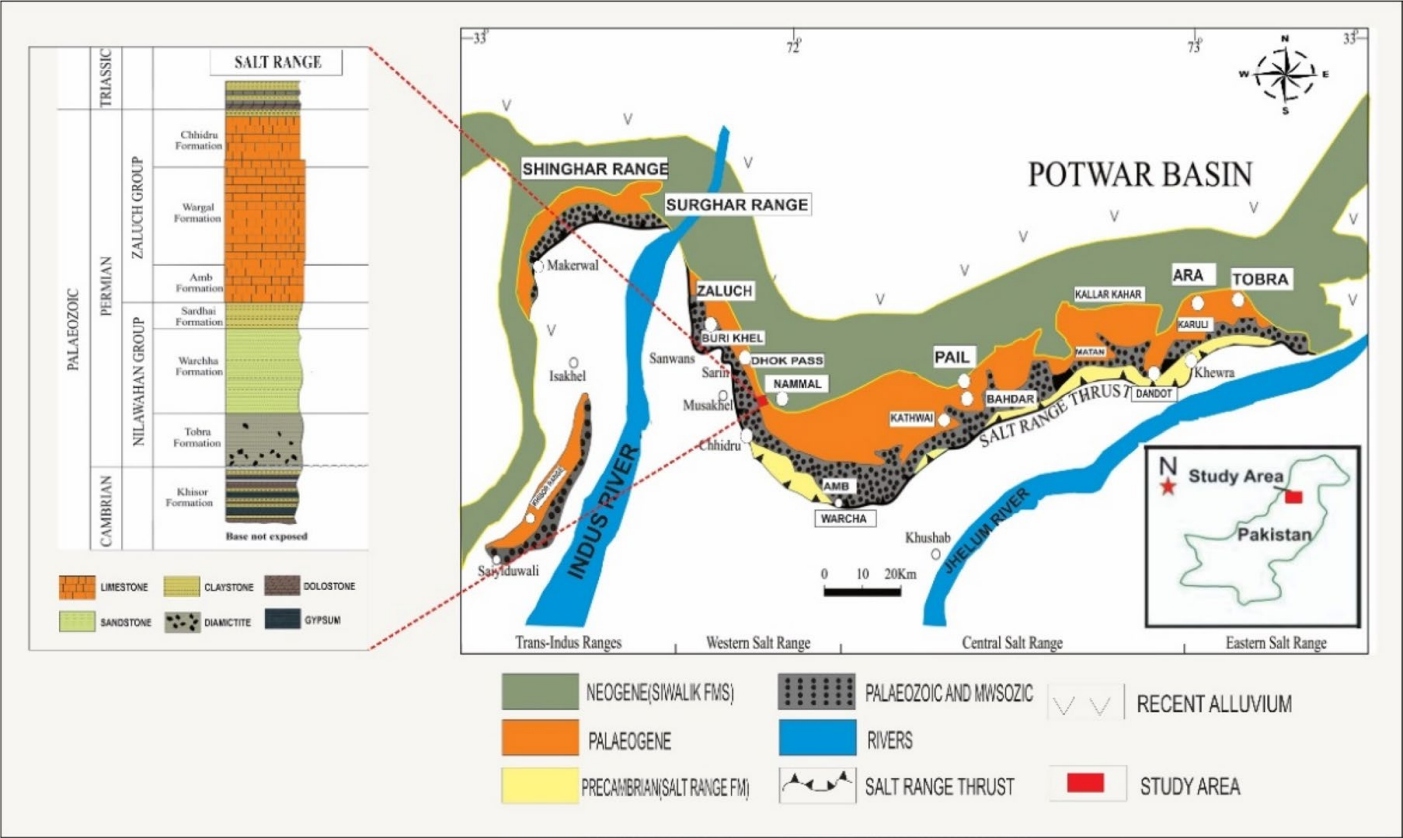
Geological and Stratigraphic setting of the study area, Salt Range, Pakistan.

Potwar Basin and Salt Range were formed by the drift of the Indian Plate towards the north and its collision with the Eurasian Plate, later on^16^. In the north-central part of the Indian Plate, the Salt Range is an active fold and thrust belt formed by the collision of the Indian Plate with the Eurasian Plate^24,25^. The Salt Range continuously experiences deformation of the compressional, transform, and extensional types^15,24,26^. The range front is characterized by the superposition of Precambrian evaporites and overlying layers atop syn-orogenic alluvium and fan material^27^. The oldest rocks in the western Salt Range originate from the Carboniferous and Permian in the Nilawahan Group, whereas the Precambrian Salt Range Formation was deposited on top of younger strata in the eastern Salt Range^28,29^ as shown in Fig. 2. Rocks in the Salt Range have been dated to various periods, spanning from the Precambrian to the Tertiary^29,30^. The Middle to Upper Permian Tethyan Zaluch Group and the Lower Permian Nilawahan Group of the Gondwanan region are separated by the Carboniferous-Permian sequence of the Salt Range Pakistan^5,31,32^.

In the Potwar Basin, Permian rocks are mainly composed of siliciclastic rocks from the Nilawahan Group and carbonate-rich rocks from the Zaluch Group. In the western Salt range, the marine siliciclastic-carbonate mixed lithofacies make up the Upper Permian Zaluch Group^33^ and it comprises of siliciclastic carbonates mixed lithofacies of Amb Formation, carbonates of Wargal Limestone and clastic-carbonate mixed lithologies of the Chhidru Formation^34^ as shown in Fig. 2.

### Wargal limestone

The Wargal Limestone displays grey and yellowish-grey colors of fresh and weathered surfaces, respectively. The Wargal Limestone is fine to medium-grained characterized by granular mosaic texture with fractures, and it is medium to thickly bedded, It is exposed along the Zaluch Nala and Nammal Gorge of the Western Salt Range and it encompasses complex lithology with the thickness of 130 m^35,36^; Fig. 2). In the Wargal Limestone, the contact with the underlying Amb Formation is well-defined and occurs at the basal sandy limestone of the formation above the topmost shale layer^36^. There reported fossils in the formation include gastropods, bivalves, and trilobites, and based on the fossils, the formation prevails throughout the middle of the Permian^36^.

### Chhidru Formation

Weathered and fresh colors of the limestone of the Chhidru Formation is grey and creamy grey, respectively and the limestone is fine to medium-grained with a granular mosaic texture having fractures, and it is medium to thickly bedded. The Chhidru Formation is in conjunction with the Wargal Limestone in a way that is both conformable and gradational. Chhidru Nala, located in the Western Salt Range, is designated as the type locality. Among the lithologies present in this area, dark grey, sandy shale is predominant at the base of the mountain range, followed by calcareous sandstone and sandy limestone above it. At the top of the Chhidru Formation lies a white sandstone layer, which is a defining aspect of the formation. Within the Salt Range, the thickest point of the rock unit measures 85 m. Several types of fossils, including those of brachiopods, gastropods, pelecypods, ammonoids, bryozoans, and fusulinids have been reported in the Chhidru Formation and based on the fauna and stratigraphy, the age of the Chhidru Formation is Late Permian^36^.

## Materials and methods

A detailed geological field and laboratory investigation was conducted on outcrop sections of Wargal Limestone and Chhidru Formation in the western Salt Range region including geotechnical and petrographic analyses. These studies have aimed to investigate Late Permian rock units and evaluate their aggregate potential for building applications. The employed geotechnical and geochemical tests were conducted for aggregate (Coarse) at the China University of Geosciences, Wuhan, China. Many Cores of 35 mm diameter with a length of 80 mm from 20 block samples (volume around 0.10 cubic feet) were drilled from the samples of limestones, collected from the outcrops to ascertain the physio-mechanical properties of the formations. In order to analyze the physical and mechanical properties of limestone, samples of crushed limestone were made into cubic forms by systematic cutting. Laboratory work comprised of several tests performed based on the standards set by the American Association of State Highway and Transportation Officials and they include point load tests (PLTs), universal compressive test, water absorption tests, aggregate porosity, specific gravity tests^37^, Los Angeles abrasion value (LAA) tests^38^, and flakiness and elongation tests following standard specifications (ASTM^39^) along with the petrography. PLT tests were carried out according to the recommendations of the International Society of Rock Mechanics (ISRM^39^) and core samples were extracted from bulk samples using a core drilling machine for unconfined compressive strength tests. For petrography, collected rock samples were thin-sectioned and around 20 thin-sections of around 0.03 mm thickness were prepared and studied under the polarizing microscope. Conventional petrography was performed using the method and chart of Scholle and Ulmer-Scholle^40^ and Hussain et al.^15^, and the grain-counting technique was used for the estimation of the mineral contents.

### Geotechnical analysis

To substantiate the aggregate application of the Wargal Limestone and Chhidru Formation, as recommended for construction projects, many coarse and fine aggregate tests must be performed under standards (ASTM C-33^39^). Therefore, some important tests on the coarse aggregates were conducted to achieve the key purpose of the research.

#### Los Angeles abrasion value (LAA)

This test aims to ascertain how well steel balls can withstand rubbing against aggregates^41^. An aggregate with a lower Los Angeles abrasion value is considered stronger than one with a higher value, and vice versa^3^. Los Angeles abrasion value was computed via the^38^ test by using Eq. (1).

1 $${\text{Los}}\;{\text{Angeles}}\;{\text{Abrasion}}\;\% = \frac{{M_{orignal} - M_{final} }}{{M_{orignal} }} \times 100$$

#### Soundness test

One of the crucial features which affects the aggregate quality is weathering. Aggregates having little change in pore volume after soaking, freezing, drying, and thawing are recommended for construction. When soundness tests are carried out on a sample, the unstable aggregates show detrimental characteristics such as map cracking, D-lines, and pop outs^5,42^. The aggregates were dried several times during the test after being immersed in Na_2_SO_4_ or MgSO_4_ solutions. The soundness of aggregates was assessed following the (ASTM C88-13)^43^ test protocol by using the Eq. (2).

2 $${\text{Soundness}}\;\% = \frac{{Initial _{mass} - Retained_{mass } }}{{Initial_{mass} }} \times 100$$

#### Specific gravity, water absorption, and aggregate porosity

The weight of an equal volume of water and an aggregate in relation to each other is expressed as their specific gravity. The amount of water a rock can absorb is determined by its ability to hold water. The concrete deteriorates as the water absorption value increases due to its expansion. Similarly, if less water is absorbed, the rock will not break down or weather. According to (ASTM C-127)^44^, aggregate specific gravities and water absorption were calculated by using Eqs. (3) and (4).

3 $${\text{S}}.{\text{G}} = \frac{1}{{{\raise0.7ex\hbox{${P1}$} \!\mathord{\left/ {\vphantom {{P1} {100G1}}}\right.\kern-0pt} \!\lower0.7ex\hbox{${100G1}$}} + {\raise0.7ex\hbox{${P2}$} \!\mathord{\left/ {\vphantom {{P2} {100G2}}}\right.\kern-0pt} \!\lower0.7ex\hbox{${100G2}$}} + \ldots {\raise0.7ex\hbox{${Pn}$} \!\mathord{\left/ {\vphantom {{Pn} {100Gn}}}\right.\kern-0pt} \!\lower0.7ex\hbox{${100Gn}$}}}}$$

4 $${\text{Absorption}}\;\% = { }\left( {{\raise0.7ex\hbox{${B - A}$} \!\mathord{\left/ {\vphantom {{B - A} A}}\right.\kern-0pt} \!\lower0.7ex\hbox{$A$}} \times 100} \right)$$

The persistence of a rock is influenced by its aggregate porosity. The main factors that affect the rock aggregate porosity are; the shape, size, and arrangement of minerals^44^. Moreover, geochemical and mechanical processes have an impact on aggregate porosity. Equation (5) was used to calculate the porosity of the aggregate.

5 $${\text{Porosity}}\;\left( {\text{n}} \right) = \left[ {\left( {1 - \frac{{W_{OD} }}{{W_{SSD} }}} \right) \times SG_{SSD} } \right] \times 100$$

#### Bulk density or unit weight

Aggregates with a higher unit weight are more compact, resulting in a reduced void ratio and greater strength^45^. Unit weight and bulk density are mainly influenced because of shape, gradation, surface roughness, specific gravity, and angularity. This test was assessed by following the (ASTM C-29)^43^ by using Eqs. (6–8) to find out the bulk density of the aggregates.

6 $${\text{Bulk}}\;{\text{density}} = \frac{Mass\;of\;aggregate\;to\;fill\;cylinder}{{Volume\;of\;measuring\;cylinder}}$$

7 $${\text{Bulk}}\;{\text{density}}\;\left( {{\text{SSD}}} \right) = ((Bulk density\{ 1 + \left( {Absorption\% } \right)/ 100)$$

8 $$\% \;{\text{Void}} = \left( {{\raise0.7ex\hbox{${\left[ {\left( {S \times W} \right) - M} \right]}$} \!\mathord{\left/ {\vphantom {{\left[ {\left( {S \times W} \right) - M} \right]} {S \times W}}}\right.\kern-0pt} \!\lower0.7ex\hbox{${S \times W}$}}} \right)$$

#### Aggregate impact value (AVI)

The relative resistance of an aggregate to a sudden shock is represented by the aggregate impact value^44^. The aggregates should be durable enough to sustain impacts without crumbling. Rocks resistant to granulation or disintegration will have a lower aggregate impact value^45^. The aggregate impact value was assessed by following the standard (BS-812)^45^ by using Eq. (10).

9 $${\text{Impact}}\;{\text{value}}\;\% = \frac{Mass\;of\;fraction\;passing\;no.7\;sieve}{{total\;mass\;of\;sample}} \times 100$$

#### Aggregate crushing value (ACV)

The aggregate crushing value depicts how resistant an aggregate crushing is to a gradually applied compressive load. Lower aggregate crushing values should be achieved to create better pavement quality, and aggregates should be able to survive crushing under load. The impact value was calculated by following the standard (BS-812)^46^ using Eq. (10)

10 $${\text{C}}.{\text{V}}\;\% = \frac{Mass\;of\;fraction\;passing\;no.8\;sieve}{{total\;mass\;of\;sample}} \times 100$$

#### Shape test (flakiness and elongation)

In the construction of bituminous and cement concrete and base course, flaky and elongated particles cause inherent fragility under large loads. To evaluate particle form in terms of flakiness and elongation, a shape test was conducted following (BS 812)^47,48^ and Eqs. (11) and (12) were used for their quantification.

11 $${\text{Flakiness}}\;{\text{Index}}\;\% = \frac{{W_{1} }}{{W_{2} }} \times 100$$

12 $${\text{Elongation}}\;{\text{Index}}\;\% = \frac{{W_{3} }}{{W_{4} }} \times 100$$

#### Unconfined compressive strength

The strength or ability to withstand applied stress by a rock is measured frequently in laboratories to choose a rock with the desired strength^49^. The UCS was calculated according to (ASTM D-7012)^50^. The test values were determined by using Eq. (13)

13 $$UCS = \frac{P}{A}$$

where P is Load and A is the cross-sectional area of the core.

#### Point load tes (PLT)

Point load strength testing is intended to be used as an index for determining the strength of rocks based on their composition and properties^51^. Various types of rock samples can be used, including cores, blocks, and irregular lumps, without preparation of specimens for this test. PLTs were performed following the International Society of Rock Mechanics' recommendations (ISRM^52^).

### Geochemical analysis

The bulk rock samples were used in the lab for crushing and powdering for the geochemical assessments. The tungsten carbide ball mill was used to grind twenty samples (three from each bulk sample) to a finer than 0.075 mm (No. 200 sieve), and the resulting powder was then sealed in polythene bags to prevent cross-contamination. The concentrations of the main oxides inside the materials were determined using the atomic absorption spectrometer (AAS) 3300, Analyst 700 with graphite furnace and mercury hydride system (MHS), with UV/VIS spectrophotometer (SP-400 UV/VIS) from Perkin Elmer, as described by Candra^50^.

### Alkali aggregates reactivity (AAR)

Concrete with aggregates containing reactive elements can react when exposed to alkali hydroxides. The reactivity can be highly dangerous only when it results in a massive expansion^53^. Due to the prevalence of reactive silica minerals in aggregates, ASR has become a more prominent reason to be considered than ACR. Aggregates of alkali-reactive carbonate have a unique composition that is not frequently found.

Reactivity of alkali-silica in concrete has been recognized since the late 1930s as a potential cause of concrete distress^51^. Carbonate aggregates are vulnerable to ACR including limestone (particularly dolomite) as well as dolostone (calcitic). Excessive aggregate growth, cracking, and de-dolomitization define the ACR reaction^54^. For the ACR reaction^54^, the underlying process Eq. (14) is used.

14 $${\text{CaMg}}\left( {{\text{CO}}_{3} } \right)_{2} + 2{\text{NaOH}} \to {\text{CaCO}}_{3} + {\text{Na}}_{2} {\text{CO}}_{3} + {\text{Mg}}\left( {{\text{OH}}} \right)_{2}$$

This procedure can swiftly determine whether or not a certain carbonate rock (limestone, dolostone, or calcitic-argillaceous-dolostone) is suitable to use as concrete aggregate by measuring its alkaline reactivity. A possible ACR could be detrimental to concrete durability if the expansion rate exhibited by a sample is higher than 0.10%^55^.

### Petrographic analysis

The Petrographic analysis is performed through the studies of thin sections under a polarized light microscope (Model Olympus BX51) to determine the depositional and chemical makeups of the rock/aggregate along with its mineralogy. To identify the reactive components in aggregates, petrographic investigations are often used^39^. Microfacies analysis was used to classify limestone of the rock units and the categorization of microfacies follows the scheme of (Dunham-1962)^55^ established for limestone.

The mineral content was calculated using a model analysis strategy. The mineralogical composition of the samples was taken into consideration since it has a significant bearing on the quality of aggregate materials^56^. Equation (15) was used to determine the percentage mineral composition.

15 $$C_{m } = \left( {\frac{{T_{m} }}{{T_{tm} }}} \right) \times 100$$

In this equation, ${C}_{m}$ is the mineral composition in percentage (%), and ${T}_{tm}$ is a Total number of counts for the entire mineral.

Using the ASTM-recommended approach and the following calculation, the aggregate porosity was calculated by using Eq. (16).

16 $$P\left( \% \right) = \left( {\frac{{W_{ssd} - W_{od} }}{{W_{ssd} - W_{water} }}} \right) \times 100$$

where P is the aggregate porosity; Wssd is saturated surface dry weight; Wod is the oven-dry weight and W is weight in water.

## Results

### Geotechnical analysis

**Figure 3:**
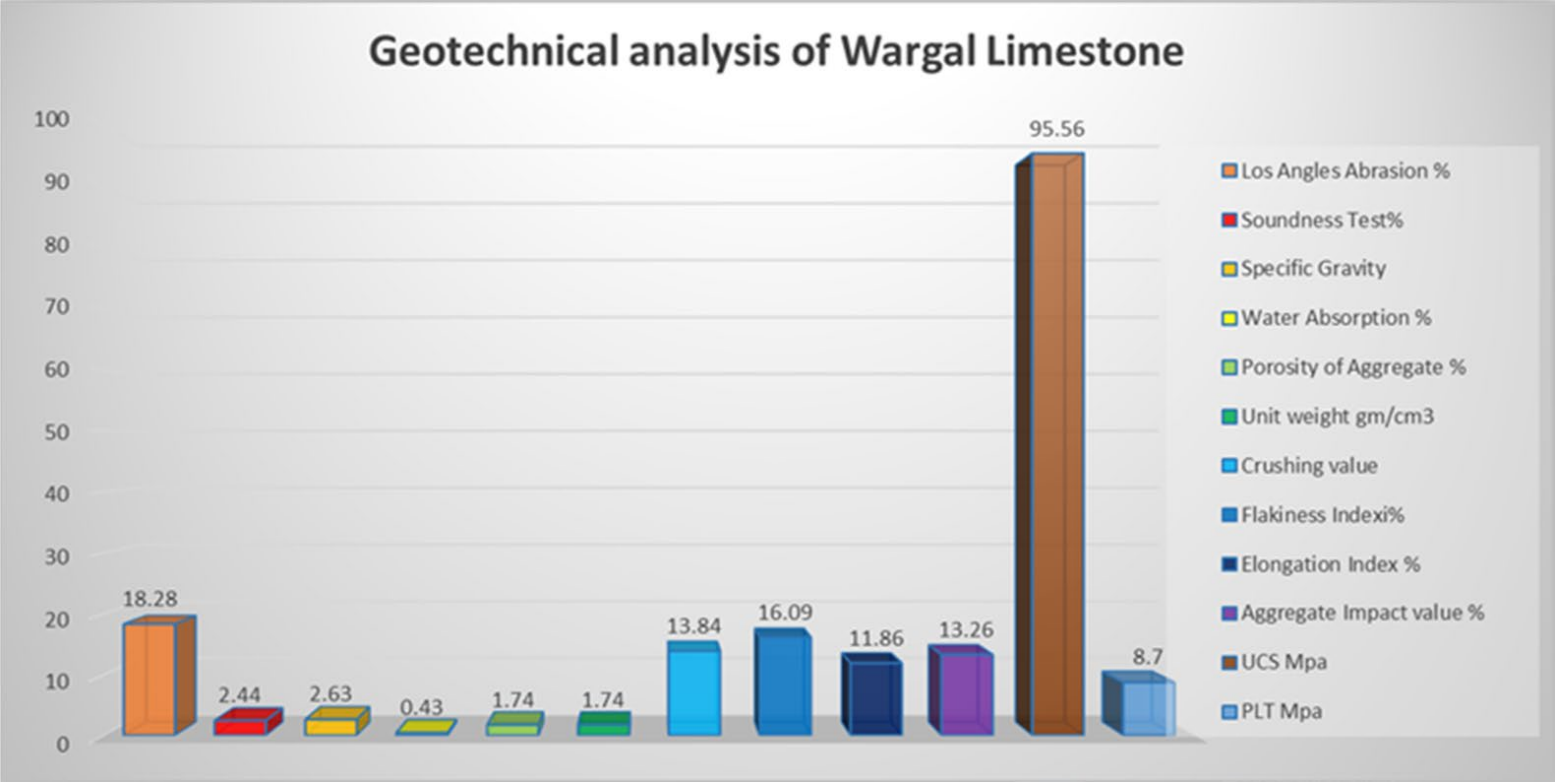
Geotechnical analysis of Late Permian Wargal Limestone, Western Salt Range.

**Figure 4:**
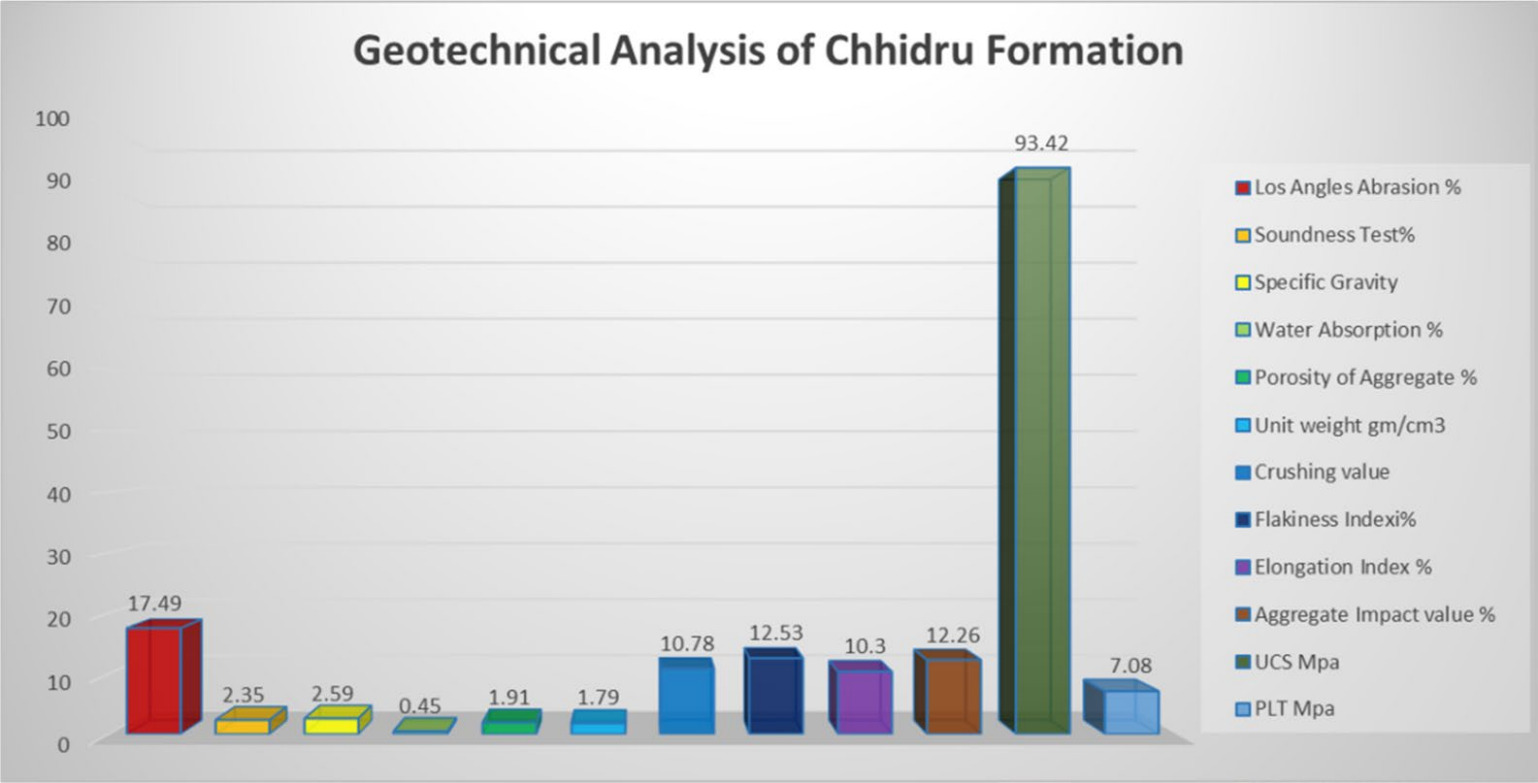
Geotechnical analysis of Late Permian Chhidru Formation, Western Salt Range.

Several integrated analyses were performed on the samples of the Wargal and Chhidru formations exposed in the Western Salt Range to elucidate the role of rock units in utilization as a geotechnical resource and especially in engineering structures. There are several factors that influence the physical properties of rocks, including the composition of the modal minerals, the cement, the grain size, and the contact between grains^52^. Similarly, the physical and petrographic attributes of sedimentary rocks have a significant influence on their mechanical characteristics^9,52,57^. Some geotechnical analyses performed are correlated to understand the physico-mechnaical properties of the rock units. The values of Los Angeles abrasion for the Wargal Limestone and Chhidru Formation are 18.28 and 17.49%, respectively (Figs. 3, 4). These values are below 40% which means that they are within the permissible limits. The recorded soundness values for Wargal Limestone and Chhidru Formation remain at 2.44 and 2.35%, respectively (Figs. 3, 4). These results indicate that the rocks of both formations are sufficiently resistant to freezing and thawing effects, with a feasibility limit of 16%.

There is a direct relationship between specific gravity and the strength of aggregate^58^ and water absorption is a direct indicator of permeability^59^. Rocks that comprise values greater than or equal to 2.55 of specific gravity are deemed acceptable for large building works^22,58^. Moreover, the minimum requirement for cement concrete is 2.60 (Naeem et al.^5^). The values of specific gravity and water absorption of the Wargal Limestone and Chhidru Formation remain at 0.43 and 0.45%, and 2.63 and 2.59, respectively (Figs. 3, 4). As per ASTM standards, the absorption capacity of these rocks is within the permissible level, i.e. 2%. In this research, the aggregate porosity values of the Wargal Limestone and Chhidru Formation are 1.74% and 1.91%, respectively (Figs. 3, 4). According to Zada et al.^16^, limestone samples from both formations bear low porosity, yet they do impart negative impacts on the mechanical properties (UCS) of a rock. The unit weight of aggregate applied in concrete tends to fluctuate from 1.20 to 1.75 g cm^3^, and the unit weight of the analyzed samples of the Wargal Limestone and Chhidru Formation are substantially within the acceptable limits of 1.74 and 1.79 g/cm^3^, respectively which can be utilized as engineering components (Figs. 3, 4).

The analyzed impact values and aggregate crushing grade of the Wargal Limestone and Chhidru Formation remain at 13.26 and 12.26%, 13.84 and 10.78%, respectively (Figs. 3, 4). Moreover, the aggregate effect values, particularly 30% are considered within the acceptable range, and the aggregate crushing values, precisely 30% are considerably below the permitted limits for utilization as an engineering material.

Particle shape affects the engineering properties of aggregates in construction by influencing material placement and consolidation^58,59^. Samples of the Wargal Limestone and Chhidru Formation comprise of index values 16.09% and 12.53% (flakiness) and 11.86% and 10.30% (elongation), respectively, and values of both formations are within the permitted limit of 40% (Figs. 3, 4).

**Figure 5:**
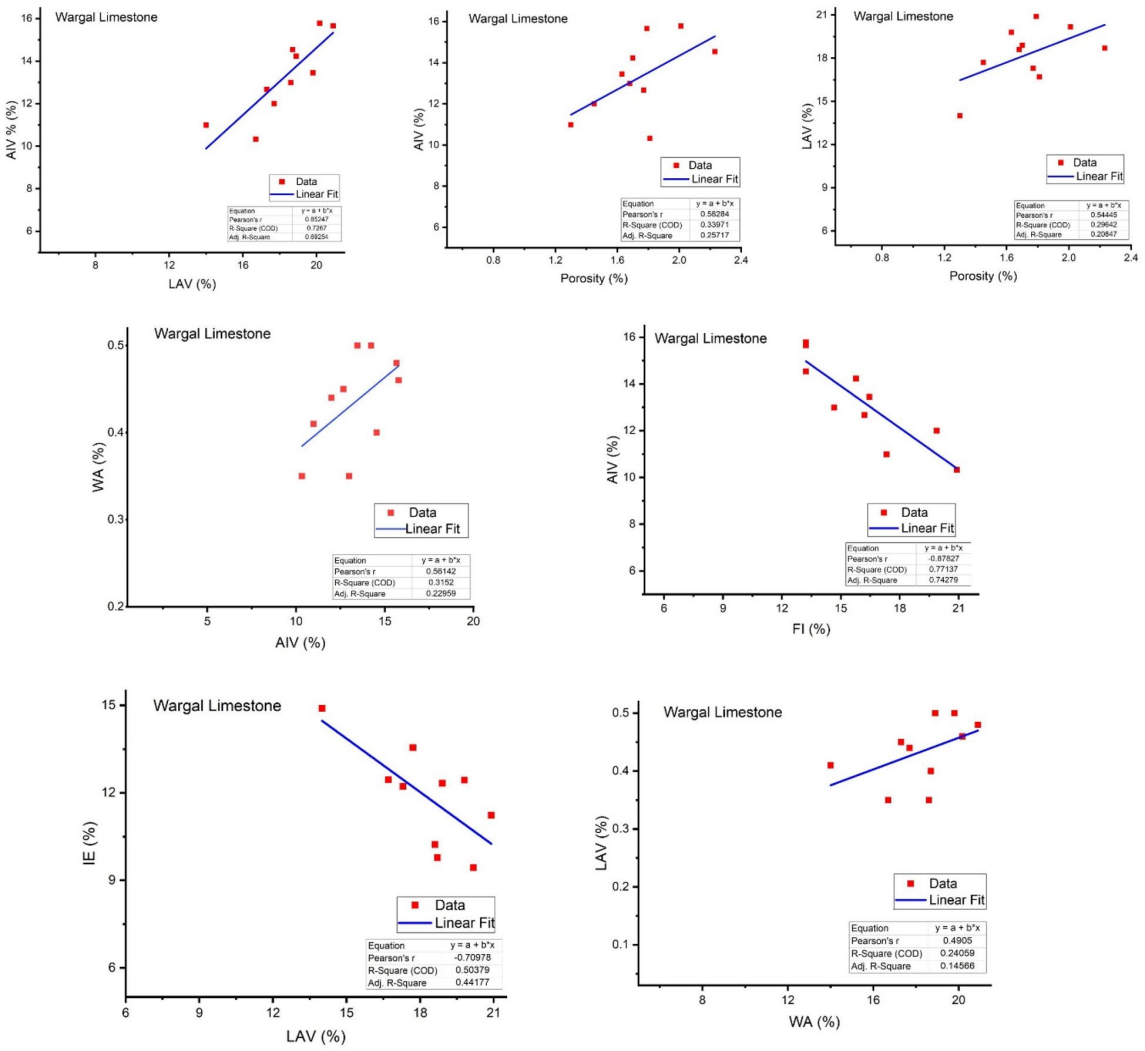
Regression analysis between Physio-mechanical properties of Wargal Limestone aggregates.

**Figure 6:**
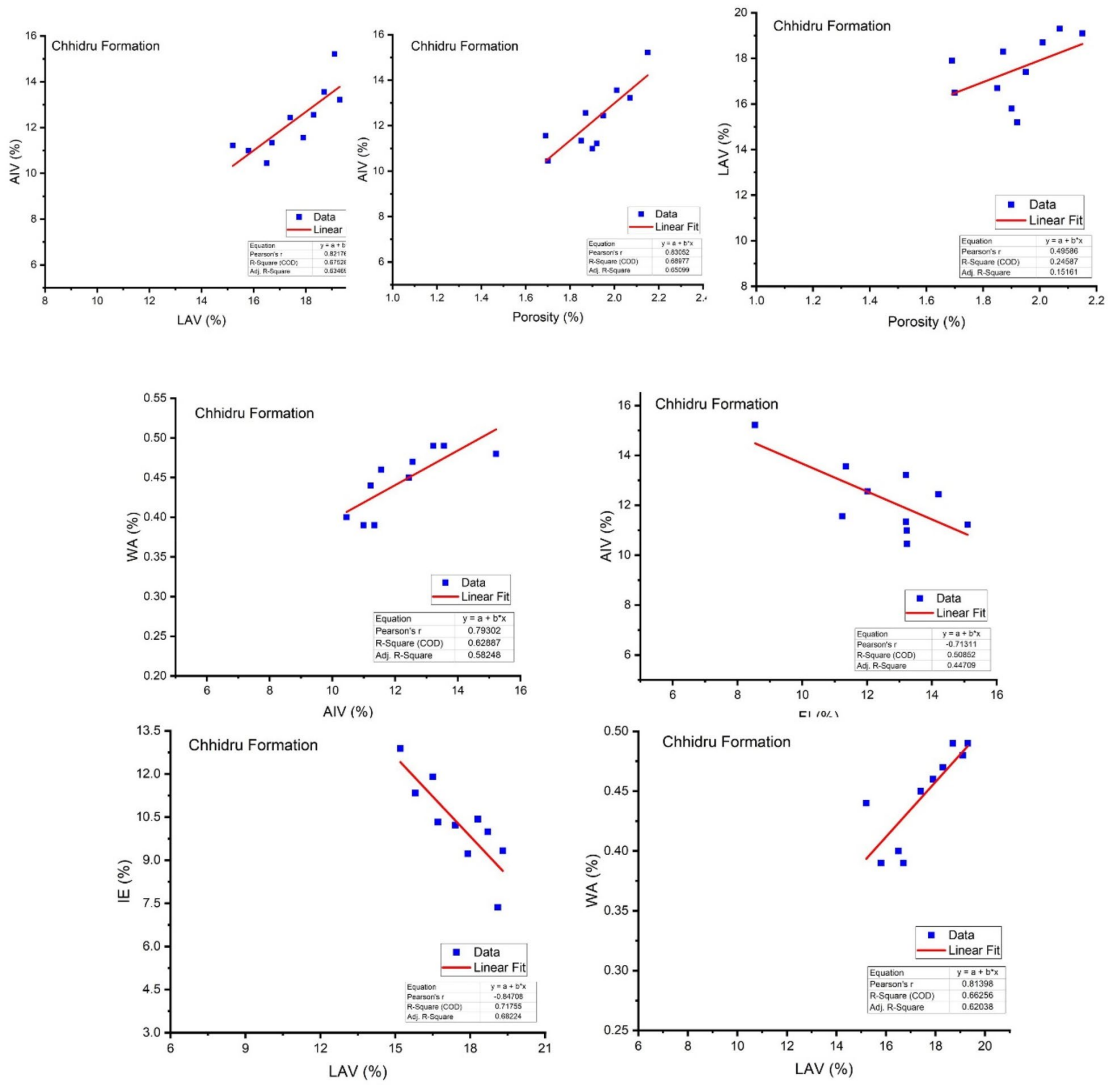
Regression analysis between Physio-mechanical properties of Chhidru Formation aggregates.

Abrasion value reflects the toughness of the aggregate under natural and stressed conditions^60^. The samples of the Wargal Limestone bear higher UCS values, i.e., greater than 95 MPa, therefore, samples of this formation can be categorized as solid rocks. The peak hardness value obtained in core samples of Wargal Limestone remains 99.2 MPa, and the lowest was 87.7 MPa, with an average value of 95.56 MPa (Figs. 3, 4). Similarly, the samples of the Chhidru Formation also have higher UCS values, i.e., greater than 95 MPa, and they can also be categorized as strong/hard rocks. The peak and lowest hardness values obtained in core samples of the Chhidru Formation are 97.2 and 89.2 MPa, respectively with a mean of 93.42 MPa (Figs. 3, 4). The Point Load test were carried out both on Wargal limestone and Chhidru formation and the results were recorded 8.7 MPa for Wargal Limestone and 7.08 MPa for Chhidru formation. The UCS and Point Laod values of both formations are within the ASTM and AASHTO prescribed standards. The regression analysis carried out in this research on the physico-mechanical properties of limestone samples from the Wargal Limestone and Chhidru Formation. The analysis revealed that there is a direct and significant relationship between Los Angeles abrasion value, water absorption, and aggregate impact value Figs. 5, 6. Additionally, a similar correlation was observed between porosity and aggregate impact value. These relationships are consistent with established standards and prior research, lending credibility to the present findings. Furthermore, the study found an inverse relationship between the flakiness index, elongation index, and aggregate impact value Figs. 5, 6. This suggests that elongated fragments have lower abrasion resistance, and the results align with previous research studies. Overall, the findings of this study provide valuable insights into the physico-mechanical properties of limestone, which could inform decisions regarding their use in construction and other applications.

### Comparison of Wargal limestone and Chhidru Formation with other limestones

According to Table.1, limestone samples in the study area can be compared with limestone samples from different well-known limestone deposits in Pakistan. Physical and strength tests on aggregates from the Wargal Limestone and Chhidru Formation yielded findings that were equivalent or parallel to those found in other investigations^5,21,23,42,61^. Aggregates with high strengths tend to have low soundness, abrasion value, impact value, crushing value, and porosity whereas lesser strength aggregates have greater soundness, Los Angeles abrasion value, aggregate crushing value, and aggregate porosity^5,62–64^. Among the studied limestones, Muzaffarabad Formation (MF) and Margalla Hill Limestone (ML) exhibited the lowest soundness values of 0.77% demonstrating higher resilience to degradation and volume alteration within the pores. However, other limestones of the formations, such as those from the Kawagarh Formation (KW), Shekhai Formation (SH), and Samana Suk Formation (SSF) showed the most desirable aggregate properties^13^, including the lowest Los Angeles abrasion values of 14.08–16.92%, and values of 11.38–14.90%, 0.25–1.04%, 1.04–2.12%, and 2.60–2.77 of aggregate impact, water absorption, aggregate porosity, and high specific gravity, respectively (Table 1). In this research study, the Wargal and Chhidru formations bear the maximum aggregate crushing values of 13.84 and 10.78%, respectively. In contrast, the recorded least value of the Wargal and Chhidru formations of the unit weight/bulk density remains at 1.74 and 1.73 g/cm^3^, respectively. The Wargal Limestone and Chhidru Formation both have the values of unconfined compressive strength values in the middle range of 95.58 and 93.90%, respectively (Table 1). Wargal Limestone and Chhidru Formation had flakiness and elongation values that were lower than those of other limestones, although nearly in the same range as SSF and KW (14.91–9.94% and 16.09–11.80%, respectively). Lockhart Limestone (LL) has the largest value of bulk density 2.70–2.78 g/cm^3^ and has a specific gravity of 2.63 and 2.59, respectively, which is fairly parallel to other limestones. On the contrary, Wargal and Chhidru formations have water absorption values of 0.39 and 0.46, respectively, which are comparable to KW, SH, SSF, WL, LL WL, and MF^5,16,23,42,61,62^. Similar both the formations have aggregate porosity values that are greater than KW, SH, and SSF and somewhat lower than ML and LL and the porosity values of the Wargal and Chhidru Formation remain at 2.02 and 2.0%, respectively. The characterizing attributes of the Wargal Limestone and Chhidru Formation include lower values of soundness, Los Angeles abrasion, aggregate impact, aggregate crushing, and water absorption due to lower amounts of bioclasts, microfractures (the presence of discontinuities like cracks, and stratification in rocks which reduce their strength). Moreover, both formations have higher specific gravity and lower aggregate porosity.

**Table 1:** Comparison of geotechnical properties of current research work those of recent studies.

Authors	Engineering Analysis	LAA %	Soundness t%	SG	WA %	Porosity %	Unit weight g/cm^3^	CV%	FI%	EI %	AIV	PLT (MPa)	UCS (MPa)
Current research	WL	18.28	2.44	2.63	0.43	1.74	1.74	13.8	16.09	11.86	13.26	8.70	95.56
	CF	17.49	2.35	2.59	0.45	1.91	1.73	10.7	12.53	10.30	12.26	7.03	93.42
Rehman et al.^21^	SSF	27.1	1.88	2.68	0.81		2.16	12.8	9.97	12.56	14.09		99.35
	KW	20.86	2.14	2.73	0.89		2.07	13.2	11.56	12.07	13.26		69.31
Naeem et al.^5^	ML	25.15		2.63	1.61	2.98			15.78	13.11	16.01		
	KW	15.85		2.77	0.69	2.12			19.26	16.42	12.71		
	LL	24.38		2.78	1.36	2.69			19.01	12.2	15.37		
	SH	16.53		2.64	0.58	1.44			15.23	28.86	14.9		
	SSF	16.92		2.66	1.04	1.61			19.01	25.2	12.08		
Ullah et al.^22^	WL	23.37	1.01	2.7	0.48		1.67	13.1	16.8	6.5	7.1		
Hussain.et al.^17^	SSF	25.18	2.97	2.74	0.33	1.73	13.13	19.55	11.55	13.93			
Asif et al.^61^	SF	22.9	1.98	2.7	0.72		1.53	22.4	12	13	14.8		99.35
	KF	19.96	1.8	2.71	0.64		1.51	19.55	15.5	12	14.48		69.31
Kamran et al.^23^	SSF	23.51	1.6	2.66	0.47		1.58		16.84	16.39	14.06	5.18	86.77
Zada et al.^16^	LL	19.6		2.7		0.9	2.34		20	23.4		4.0–9.9	21.21–86.61

WL, Wargal Limestone; CF, Chhidru Formation; SSF, Samana Suk Formation; KW, Kawagarh Formation; LL, Lockhart Limestone; KL, Kohat Limestone; SH, Shekhai Formation.

### Geochemical analysis

The results of the geochemical analysis (Tables 2 and 3) indicate that the samples of the Wargal Limestone comprised concentration of SiO_2_, TiO_2_, Al_2_O_3_, Fe_2_O_3_, MnO, MgO, CaO, Na_2_O in ranges of 1.99–2.65, 0.01–0.1, 0.65–1.01, 0.32–0.66, 0.001–0.008, 2.21–3.01, 62.34–70.12, 0.59–0.74, respectively. Likewise, these concentration ranges in the samples of the Chhidru Formation consist of 4.5–13.64, 0.033–0.077, 0.63–0.99, 0.55–0.76, 0.002–0.007, 2.01–2.89, 61.32–64.33, 0.21–0.55, respectively. Samples from the Wargal Limestone and the Chhidru Formation were taken with mean CaO (Calcium oxide) values of 57.12 and 50.53%, respectively (Table 2).

**Table 2:** Geochemical analysis of the Wargal Limestone for key oxides.

No	CaO (%)	MgO (%)	Na_2_O (%)	Fe_2_O_3_ %	MnO (%)	SiO_2_ (%)	TiO_2_ (%)	Al_2_O_3_ (%)	L.O.I (%)
W-01	57.1	2.21	0.74	0.32	0.001	1.99	0.01	0.65	36.7
W-02	58.9	2.56	0.89	0.45	0.005	2.33	0.04	0.76	33.4
W-03	55.3	2.99	0.67	0.66	0.003	3.1	0.09	0.99	35.6
W-04	57.2	3.01	0.59	0.36	0.008	2.65	0.1	1.01	34.2
Avg	57.125	2.6925	0.7225	0.4475	0.00425	2.5175	0.06	0.8525	34.975

**Table 3:** Geochemical analysis of the Chhidru Formation for key oxides.

N0	CaO (%)	MgO (%)	Na_2_O (%)	Fe_2_O_3_ %	MnO (%)	SiO_2_ (%)	TiO_2_ (%)	Al_2_O_3_ (%)	L.O.I (%)
CH-01	54.2	2.01	0.34	0.55	0.003	4.5	0.077	0.99	35.4
CH-02	49.3	2.13	0.21	0.73	0.004	13.65	0.033	0.73	33.1
CH-03	48.7	2.67	0.45	0.76	0.007	11.67	0.065	0.63	31.4
CH-04	49.2	2.89	0.55	0.69	0.002	12.6	0.043	0.76	32.5
Avg	50.35	2.425	0.3875	0.6825	0.004	10.605	0.0545	0.7775	33.1

In this research, the proportion of silica in the samples of the Wargal Limestone stays extremely low, with an average value of 2.52% (Table 2), whereas the proportion of silica in the samples of the Chhidru Formation remains far greater than the Wargal Limestone, comparatively, with an average value of 10.60% (Table 3). Increase in the content of alumina in concrete increases the rate at which it absorbs water, which in turn increases the rate at which evaporation occurs throughout the concrete hardening, leading to an increase in cracks and, ultimately, a deterioration of the concrete occurs^65^. Consequently, alumina is considered an impure element in carbonate rocks. The average concentration of alumina in samples from the Wargal Limestone, as well as the Chhidru Formation, have 0.8% and 0.77%, respectively, however, this concentration would not affect the durability of the concrete. The ACR may become more severe if there is a large percentage of magnesium in the form of the mineral’s dolomite^66^. Samples of the Wargal Limestone and Chhidru Formation bear low mean values of magnesium oxide of 2.69% and 2.42%, respectively. The calcium content of the samples also contributed to the slightly higher Loss on ignition (LOI) values with average values of 25.29 and 20.80% from the Wargal and Chhidru formations, respectively. The geochemical findings of the Wargal Limestone and Chhidru Formation are found consistent and well matched with the geochemical analysis of previous research on aggregates.

Elçi et al.^67^ proposed an equation (Eq. 17) for determining the chemical homogeneity of limestone, stating that homogeneous limestone has a chemical homogeneity of more than or equal to 95.

17 $$Chemical\;homoginity = 100 - {\text{Sio}}_{2} \% + \left( {{\text{Al}},{\text{Fe}}} \right)_{2} 2{\text{O}}_{3}$$

According to Eq. (17), the Wargal limestone is homogeneous limestone with a CaCO_3_ content more than 95%, whereas the Chhidru formation is heterogeneous limestone with a CaCO_3_ content less than 95%.

**Figure 7:**
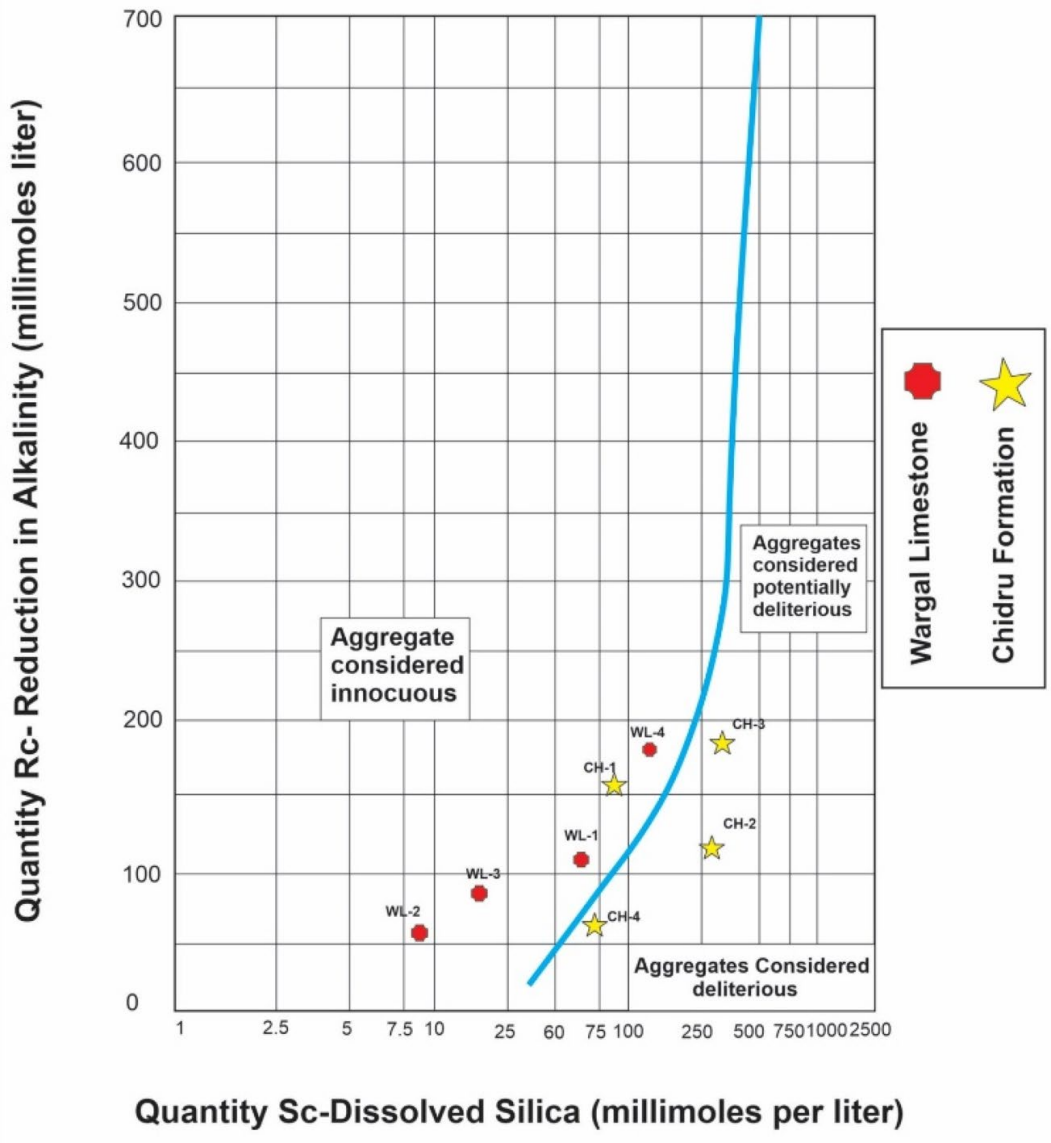
The Innocuous nature of Wargal Limestone and Chhidru Formation.

The average results (from the triplicates) of the dissolved silica (Sc) and reduction in alkalinity (Rc) were obtained by the chemical method for the different aggregates (Tables 1, 2). The Sc of the aggregates of the Chhidru Formation has high values of dissolved silica, representing a higher content of silica mineral in an amorphous structure of aggregates of the Chhidru Formation. The amorphous silica mineral has a highly disordered structure which makes it unstable at high pH conditions. On the other hand, the aggregates of the Wargal Limestone bear low values of dissolved silica, reflecting a lower content of an amorphous silica mineral. A graph comprised of mean Sc and Rc values was plotted following the (ASTM C 289) chemical test (adapted from ASTM C 289^68^), which illustrates the division between innocuous and deleterious aggregates (blue solid curve). Based on the correlations between Sc and Rc, an innocuous behavior was found for aggregates of Wargal Limestone, whereas the aggregate of the Chhidru Formation stays in the deleterious field as shown in Fig. 7.

**Figure 8:**
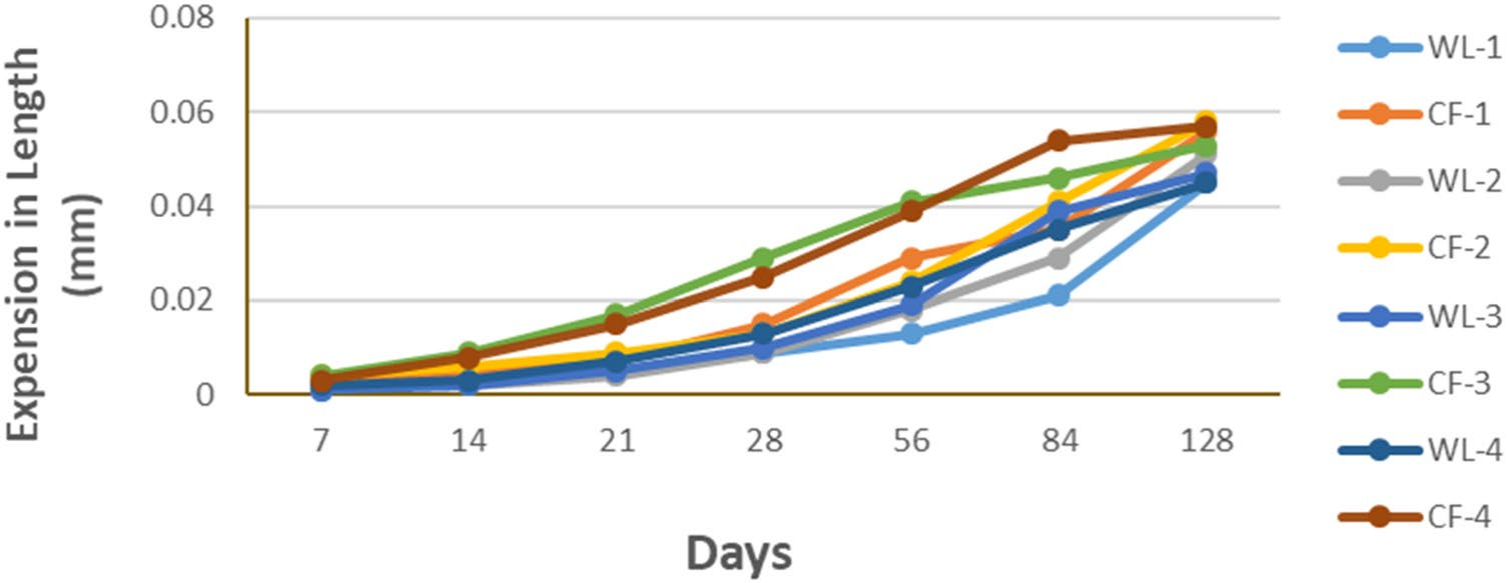
The change in length due to ACR reactivity in Wargal Limestone and Chhidru Formation.

The aggregate of the Chhidru Formation presents the highest value of Rc (197.40 mmol/L) and the aggregate of the Wargal Limestone shows the lower value (191.20 mmol/L). The determined Sc values for the Chhidru Formation range from 73.5 to 370.5 mmol/L representing the highest value, whereas 8.5 to 165.66 mmol/L values recorded in the Waragal Limestone are the lowest. This is also confirmed by the test results which depict that only samples of the Chhidru Formation are found deleterious for their reactivity to alkalis and the remaining samples (Wargal Limestone) were classified as innocuous. Moreover, in terms of the ASR, the samples of the Wargal Limestone also illustrate that the aggregates from this rock units are innocuous and thus have no deleterious effect as shown in Fig. 7. However, no major major risk of ACR is obvious for the aggregate of the Chhidru Formation as well. If the expansion values of the test sample are less than the 0.10% limit established by the (ASTM C-586)^69^ standard, as depicted in Fig. 8, then it is possible that they do not contain any ACR. According to Fig. 8, which illustrates alkali carbonate reactivity, the tested samples could be clean of ACR because their expansion values are lower than the 0.10% criterion established by the (ASTM C-586) standard^69^. Recorded loss on ignition (LOI) values stay at 20.1–29.65% and 19.9–25.73% in samples of the Wargal and Chhidru formations, respectively.

### sPearson correlation analysis among major oxides

Other than the relationships on the physico-mechanical properties, the correlation has been made for the major oxides as shown in Tables 4, 5. The correlation coefficient analyses show a strong positive association between LOI and CaO in Wargal limestone, which is an indication of pure limestone, nonetheless, there is a weak negative correlation between L.O.I and CaO in the Chhidru formation, indicating that the Chiddru formation's limestone is not pure and falls into hetrogenous limestone. Furthermore, LOI and CaO exhibit adverse relationships with SiO_2_. The greater calcite and CaO content improves the aggregate's strength and durability and conforms with the worldwide standards required by the cement industry^65^.

**Table 4:**
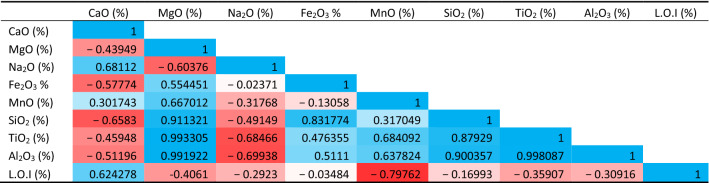
Pearson correlation analysis among major oxides for the Wargal Limestone.

**Table 5:**
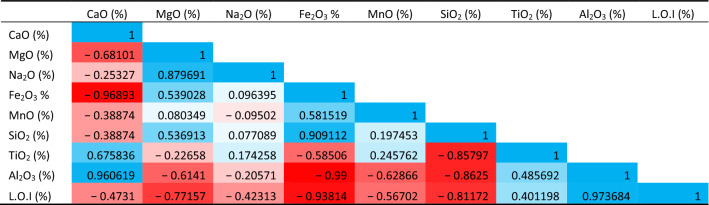
Pearson correlation analysis among major oxides for the Chhidru formation.

The fact that CaO (from calcite) and SiO_2_ (from quartz) have a negative correlation indicates that these two mineral phases are unrelated and come from separate mineral phases. Because calcite's carbonate component accounts for a large portion of LOI, there is a significant positive link between LOI and CaO concentration. Additionally, it exhibits a negative connection with the SiO_2_ table (Table 5).

### Petrographic analysis

**Figure 9:**
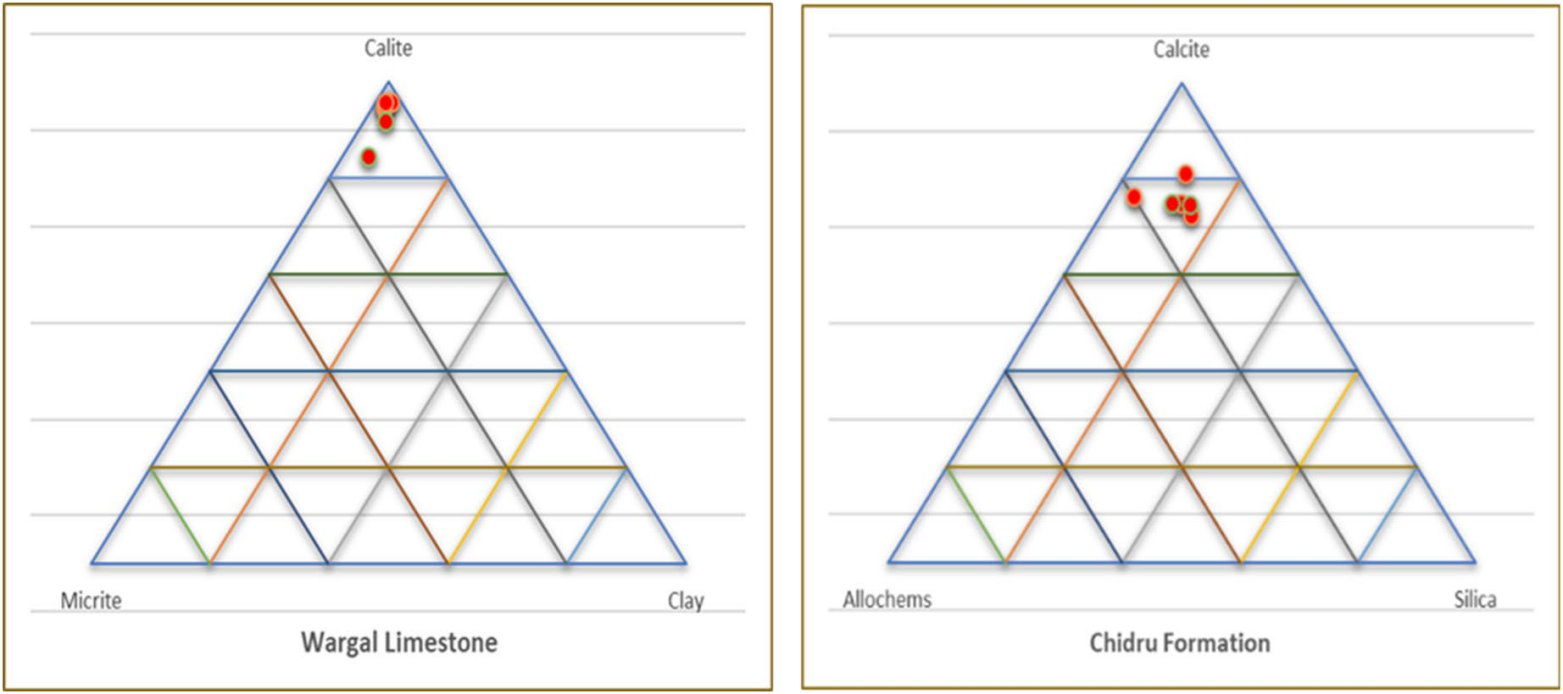
Ternary Plots diagram shows the classification of Wargal Limestone and Chhidru Formation.

Based on petrography and microfacies analysis, lithofacies of the Wargal Limestone and Chhidru Formation are categorized into mudstone, wackestone, and wackestone and floatstone, respectively. Petrography revealed that the samples of the Wargal Limestone are characterized predominantly by calcite followed by microfossils with minor quantities of clay, quartz, chalcedony, and dolomite. In the Wargal Limestone, calcite concentrations varied from 90 to 95%, micrite was 4–8%, clay remained 2–3% and concentrations of other minerals, such as pyrite, limonite, and hematite were found in traces. Discoloration along micro-fractures was a telltale sign of iron leaching which prevailed in some samples of Wargal Limestone with rare iron-bearing stylolites and veins. The stylolites reflect that the rocks had been chemically compressed, maybe as a result of overburden pressure and/or tectonic stresses. The Chhidru Formation is classified into wackestone and floatstone microfacies and it is typified by the main constituents of calcite, and micrite with minor amounts of quartz and allochems (Fig. 9). The concentration of the feldspar and quartz minerals remains in the range of 2–15%, allochems abundance ranges from 6 to 20%, and calcite accounts for 71–81%. The quartz content of the Chhidru Formation ranges from 2 to 15%, making it potentially reactive to use in concrete, According to Ramsay^70^. If the microcrystalline quartz amount in aggregate is larger than 5%, it will be hazardous for concrete usage; consequently, the Chhidru Formation aggregate should be utilized with care as concrete material.

**Figure 10:**
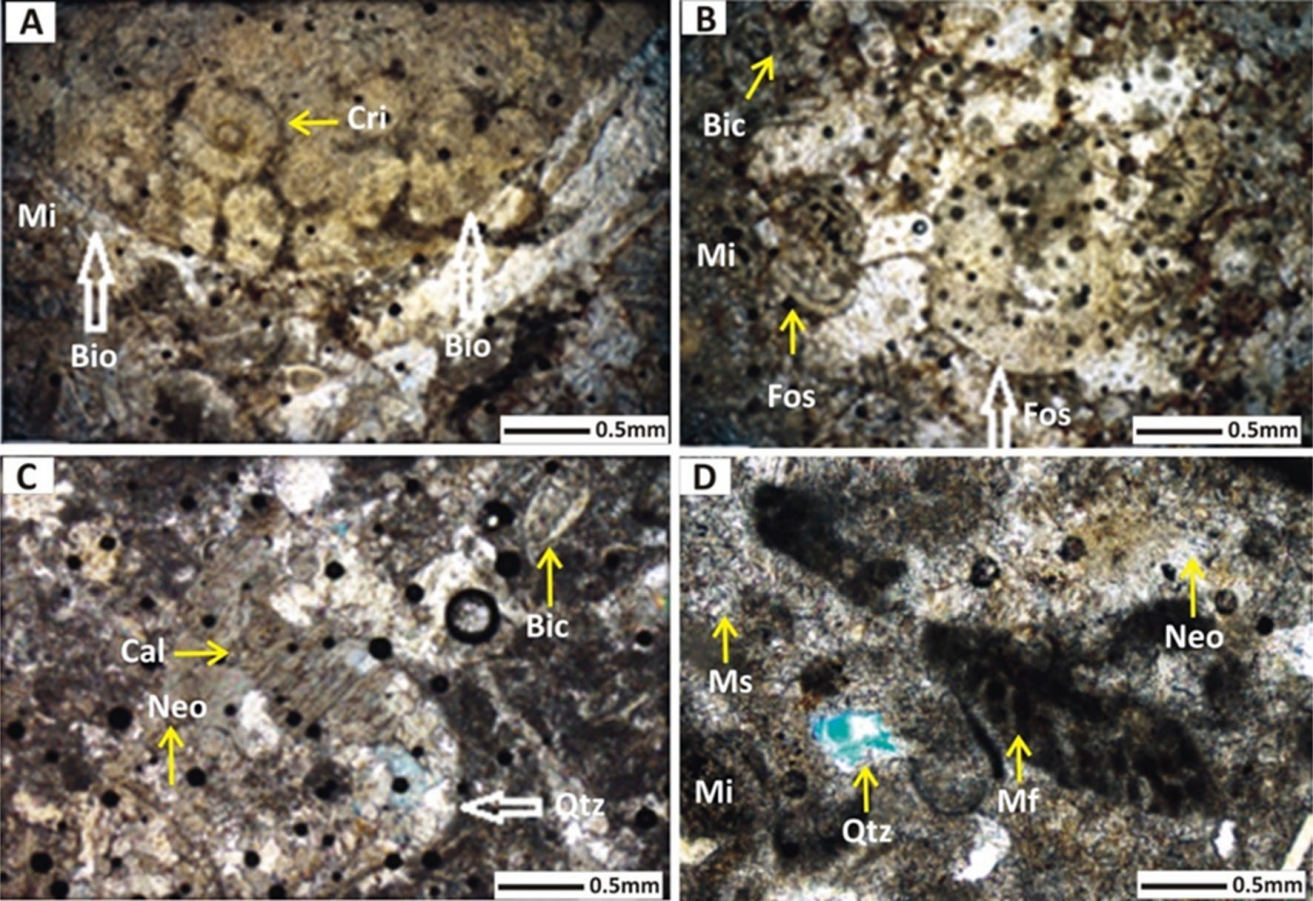
Microphotos of the Wargal Limestone exposed in the Nammal Gorge section of Western Salt Range. (A) Represents mud-wackestone microfacies where bold arrows show bioturbation, and crinoid fossil (Cri) and lime mud or micrite (mi). (B) Also illustrates mud-wackestone microfacies, whereas bold and line arrows represent undifferentiated fossil (Fos) and other features including micrite (Mi) and bioclast (Bic). (C) Represents wackestone microfacies where quartz (Qtz) is shown by a bold arrow and calcite twinning (Cal) can be seen in the middle along with bioclast (Bic) and neomporhism (Neo) diagenetic feature. (D) also outlines the wackestone microfacies represented by micritized fossil (Mf), neomorphism (Neo) and microsparite (Ms), and micrite (Mi).

**Figure 11:**
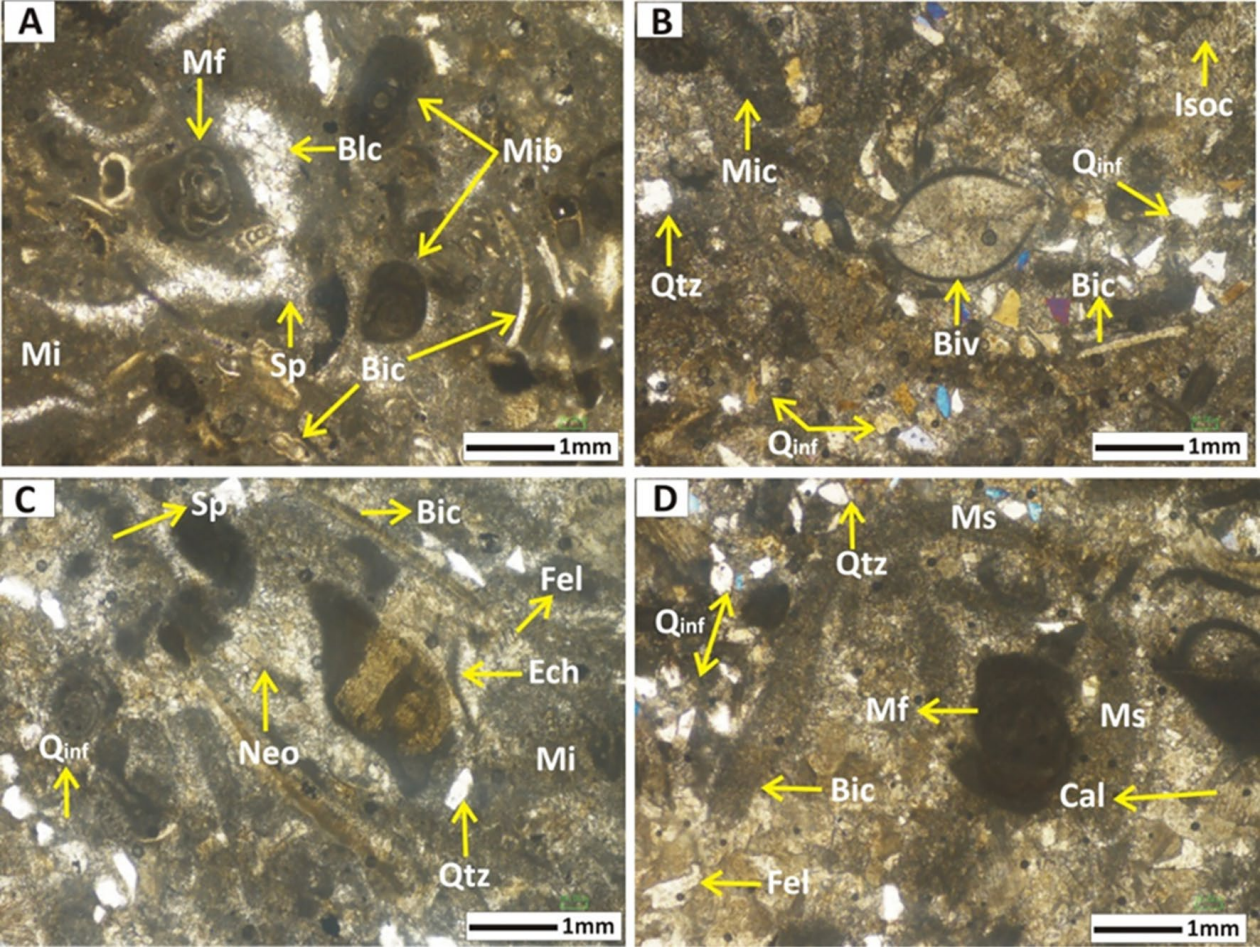
Representative micro-photos of the Chhidru Formation representing the allochemical and orthochemical abundances in a wackestone and floatstone microfacies; wherein (**A**) allochems are shown by micritized fossil (Mf), micritized bioclasts (Mib) and bioclasts (Bic), whereas orthochems are shown by blocky cement (Blc), micrite or mud (Mi) and sparite cement (Sp). (**B**) represents bivalve fossil (Biv), bioclast (Bic) as allochems, cement including isopachous cement (Isoc), micritic cement (Mic), and some quartz grains (Qtz), and quartz influx (Qinf) in the micritic matrix or mud. (**C**) shows the fossil echinoderm (Ech), bioclast (Bic), micrite matrix (Mi), sparite cement (Sp), neomorphism diagenetic feature (Neo) with embedded quartz (Qtz, feldspar (Fel), and quartz influx (Qinf). (**D**) Represents micritized fossil (Mf), bioclast of a brachiopod (Bic), with microsparite cement and minerals of quartz (Qtz), calcite (Cal), feldspar (Fel) and the quartz influx (Qinf).

It was revealed in petrography that both the rock units bear lower porosity (Figs. 10, 11). Values of the porosity range from 0.8 to 0.99% in Wargal Limestone and 0.40 to 0.77% in the Chhidru Formation. However, the fabric features of the microfacies, such as microfractures, bioclasts, and stylolites have a significant role in causing the enhancement of the porosity in the sample. This is true for the Wagal Limestone which bears higher porosity than that of the Chhidru Formation, comparatively.

### Influence of petrographic characteristics on engineering properties

**Figure 12:**
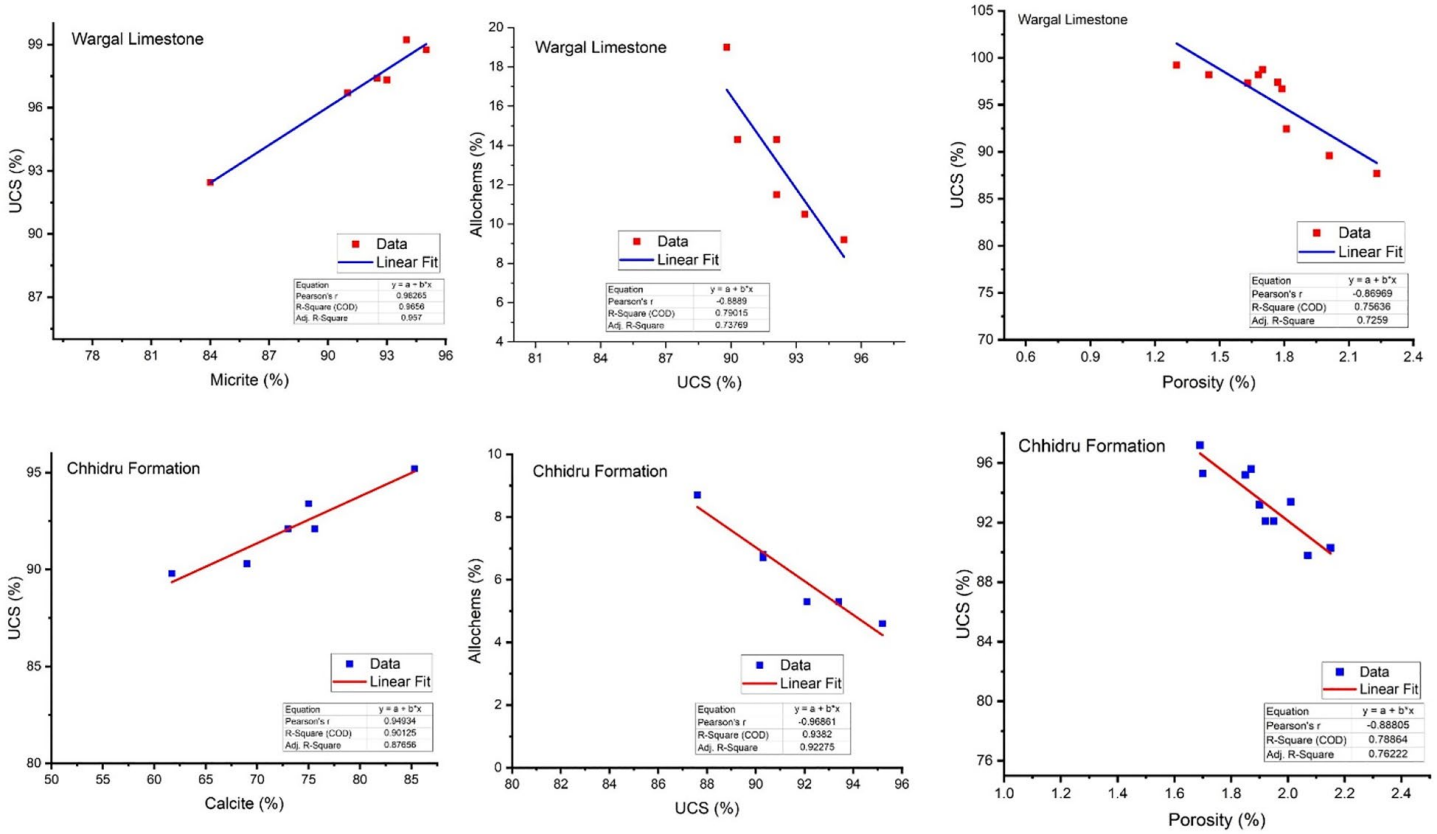
Correlation between Petrographic content and engineering characteristics (UCS) of Wargal Limestone and Chhidru Formation.

**Figure 13:**
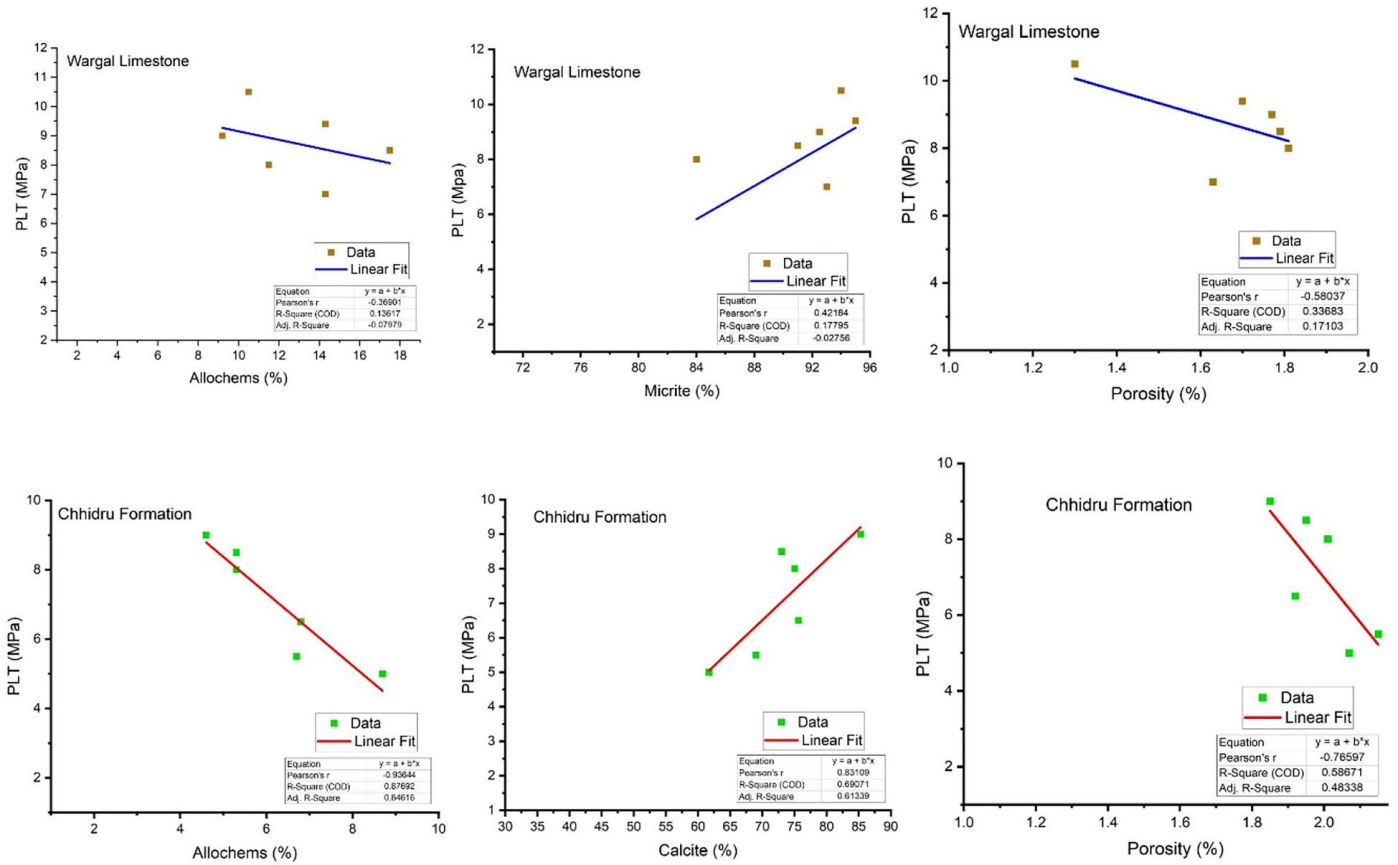
Correlation between Petrographic content and engineering characteristics (PLT) of Wargal Limestone and Chhidru Formation.

The data about physico-mechanical aspects and petrography were plotted into a regression analysis for examining the interconnections between the petrographic and engineering features (Figs. 12, 13). According to Ramsay^70^, Hartley^75^, and Lees and Kennedy^76^, petrographic characteristics and microstructure influence aggregate characteristics, and the petrographic and textural features of rock control its mechanical characteristics, Therefore, the effects of the petrographic contents on engineering properties are very important for the suitability of aggregate sources. The objective of the regression analysis is to minimize the squared deviations of measured points from the fitted line, which was calculated via the points. Calculations were also made to determine the fitted lines' equations and coefficients of determination (R2). Regression models explain most of the variability in y when (R2) is close to 1. As long as the *p*-value is less than 0.05, it is considered that the model is significant^71^. Figures 12, 13 show a correlation between the petrographic properties (calcite, bioclasts, and porosity) and the characteristics of strength (UCS) and the Point load test (PLT).

A significant linear relationship prevails between calcite concentration and strength characteristics (UCS) and Point load test (PLT) of Wargal Limestone (R2 = 0.96), (R2 = 0.18) and Chhidru Formation (R2 = 0.90), (R2 = 0.69) respectively (Figs. 12, 13). However, an inverse but significant relationship (*p* ≤ 0.05) is observed between bioclasts and strength properties (UCS) and (PLT) of Wargal Limestone (R2 = 0.79), (R2 = 0.14) and Chhidru Formation (R2 = 0.94), (R2 = 0.87), respectively. Similarly, an inverse relationship but significant (*p* ≤ 0.05) between porosity and strength properties (UCS) and (PLT) of Wargal Limestone (R2 = 0.80), (R2 = 0.33) and Chhidru Formation (R2 = 0.94), (R2 = 0.58), respectively. The same characteristics were compared by Naeem et al.^5^, who worked on Margalla Hill Limestone (ML) and Lockhart limestone (LL) in the Rumli region of Islamabad, Pakistan, and Asif et al.^61^, who worked on Eocene carbonates for utilizing in engineering structures. Moreover, Zada et al.^16^ and Kamran et al.^23^ made the same correlation for aggregates and attained the same significant results. In contrast to the current investigation, a comparable positive relationship between UCS and calcite and PLT and Calcite contents exists, and an inverse relationship between UCS and bioclasts and porosity is observed. Similarly, when examining the physicomechanical properties of aggregates for construction purposes, SYA Shah et al.^10^ discovered an inverse association between UCS and porosity. According to our analysis, the obtained model for the correlation between the UCS and the petrographic content, as well as the PLT and the petrographic content, is statistically significant, and the p value is less than 0.05.

The correlation between petrographic constituents and mechanical parameters was low to moderate in all cases. In order to determine the mechanical response of each sample from the Wargal Limestone and Chhidru Formation aggregates, we plotted the UCS and PLT values against the cumulative percentage of micrite and calcite, allochems, and porosity. These results depict similar variation in mechanical parameters that directly corresponds to the cumulative percent proportion of micrite/calcite. In contrast, mechanical characteristics have an inverse relation with cumulative percentage porosity and allochems.

WA and LAV have a significant positive relationship (Figs. 5, 6) and WA bears a direct relationship with AIV (Figs. 6, 7) which indicates that substantially more porous aggregate is more prone to crumbling, resulting in poorer bearing resistance for the material. In such limestones, there is a significant negative association between both AIV and IF (Figs. 5, 6), which is in accord with the results of Zarif et al.^72^.

Porosity is intrinsically related to AIV (Figs. 5, 6) which indicates that if porosity increases, the resistance to mechanical impact also augments. Similarly, the porosity of aggregate is adversely linked to the strength (UCS) of the material. The presence of voids affects the strength of aggregate due to stress concentration around these voids^62^. Significant direct correlations or relationships between porosity and LAV of all carbonate aggregates are observed (Figs. 5, 6) which demonstrate that an increase in porosity results in the reduction of abrasion resistance (greater LAV). A considerable positive relationship exists between LAV and AIV (Figs. 5, 6), both of these strength characteristics fluctuate immediately, as per previous studies^72^. An inverse relationship between LAV and IE (Figs. 5, 6) depicts lower abrasion resistance of elongated fragments.

## Discussion

The physical properties of the selected samples from the late Permian carbonates (Wargal Limestone and Chhidru Formation) were determined through tests of Los Angeles abrasion, soundness, Specific gravity, water absorption, porosity, unit weight, Aggregate crushing and Aggregate impact values, flakiness and elongation value and tests of UCS and PLT. The results of the Los Angles abrasion and soundness tests reveal that the rocks from both formations have enough resistance to freezing and thawing effects and are feasible within the range of permissible limits for mega construction projects. According to calculations involving bulk density, specific gravity, water absorption, and porosity, there is no chance for water to penetrate limestone and, hence, cause damage to model structures^73^. This finding explains that the surface of building materials with a low degree of water absorption capacity and porosity will be affected negligibly or not at all by weathering agents, such as wind or rainfall. Furthermore, the cumulative elongated and flaky index values are within the safe range defined for road construction.

The regression analysis between physico-mechanical properties of the limestones of the Wargal Limestone and Chhidru Formation showed that the relationships between Los angles and Water absorption and Aggregate impact value, porosity and Aggregate impact value, and porosity and Los angles abrasion value are direct and correlatable and such relationships are in accord with the defined standards and previous studies^73^. Similarly, an inverse relationship between the Aggregate impact value and the Flakiness index, as well as the Elongation index, is observed which depicts lower abrasion resistance of elongated fragments, and the results are also matched with the previous research studies^74^.

Based on the mineralogical and geochemical analyses, the limestone samples of both formations are mostly composed of the mineral calcite and lime mude i.e. micrite qualifying the international standards required for cement manufacture as shown in the previous studies of Shah (Yasir et al.^10^), (Naeem et al.^5^), (Asif et al.^61^), (Kamran et al.^23^) and (Zada et al.^16^). Pearson's correlation analysis revealed positive relationships between CaCO3, CaO, and LOI. Moreover, the ASR test demonstrated that the Wargal Limestone is adequate for use as an aggregate material in small and major construction projects, while the sample from the Chhidru Formation does not qualify for use in concrete due to the higher concentration of Alkali silica and should be used with caution, as previous research by Malahat et al.^73^ also added that the aggregates containing high silica content are not qualified for concrete due to high expansion rate. Moreover, as a result of high CaCO_3_ concentration, low dolomite, and silica concentrations there are detrimental effects on alkali-aggregate reactions, such that ACR and ASR WA and LAV have a significant positive relationship (Figs. 5, 6) and WA bears a direct relationship with AIV (Figs. 6, 7). This indicates that substantially more porous aggregate is more prone to crumbling, resulting in poorer bearing resistance for the material. In the limestone samples of the Wargal Limestone and Chhidru Formation, there is a significant negative relationship between both AIV and IF (Figs. 5, 6), which is in accord with the results of Zarif et al.^72^.

According to the petrographic analysis carried out in this research, the percentage of calcite increases the overall strength of the limestone, whereas the percentages of porosity and bioclasts decrease the mechanical properties of the limestone by reducing the values of UCS and PLT. The samples with the largest porosity have the lowest UCS and PLT values, whereas those with the lowest porosity have the highest UCS and PLT values. The results clearly show that the higher the porosity and bioclast content, the lower the strength (UCS and PLT) of the limestone sample will be, whereas a higher amount of calcite or micrite will increase the strength and stability of the limestone sample. According to Zada et al.^16^, the greater abundance of calcite or micrite leads to an increase in the stability and strength of the rock and higher bioclast contents result in relatively weak mechanical properties (UCS). Similarly, they also added that the higher porosity results in imparting lower strength (UCS) relatively. Based on the petrography, after following the ASTM (C 295-12)^75^ guidelines, the Wargal Limestone bears no potentially harmful mineral making the studied rock unit favorable for construction and as an aggregate source for roads and bridges. On the other hand, the limestone of the Chhidru Formation to use as an aggregate source, and extra care is required to utilize it for the concrete material. The recent studies of Zada et al.^16^, Asif et al.^61^, and Kaybasi et al.^76^ also revealed the same results in their studies.

Overall, the characterizing attributes of the Wargal Limestone and Chhidru Formation include lower values of soundness, Los Angeles abrasion, aggregate impact, aggregate crushing, and water absorption due to lower amounts of bioclasts and microfractures. Moreover, both formations have higher specific gravity and lower aggregate porosity. Therefore, based on petrographic, geochemical, and geotechnical analyses, the Wargal Limestone can be deemed appropriate as a broad natural source for roads, concrete, and other geotechnical and engineering applications On the contrary, the aggregates of the Chhidru Formation should be used with extra care in concrete construction projects due to the existence of certain deleterious contents, comparatively.

## Conclusion

In this research, the geochemical, petrographic, and geotechnical properties of the Late Permian Wargal Limestone and the Chhidru Formation in the Western Salt Range were explored to assess their suitability as a potential source of construction aggregate. The geochemical and petrographic analyses of the Wargal Limestone exhibited no deleterious substances that would have caused alkali-aggregate reactions. However, the geochemical and petrographic investigations of the Chhidru Formation revealed some deleterious material that may cause an alkali-aggregate reaction; as a result, the aggregates of the formation should be utilized in concrete with more caution. The physicomechanical test results for Wargal Limestone and Chhidru Formation are well within the various international standards, and can therefore be strongly recommended for the construction of engineering/geotechnical structures. The results obtained from the laboratory experiments of the studied rock units for evaluating their physical and mechanical properties were analyzed using simple statistical regressions. To determine whether or not they matched the criteria as a source of aggregate for the construction industry, the values of various physical characteristics were compared with the standards of BS and ASTM. The relationships between CaCO_3_, CaO, and LOI have manifested a robust positive relationship between these three variables, based on Pearson's correlation method. The relationship between petrographic and physicomechanical characteristics indicated that the UCS and PLT are directly related to the calcite contents and inversely linked to the porosity and bioclasts. Therefore, based on petrographic, geochemical, and geotechnical analyses, the Wargal Limestone can be deemed appropriate as a broad natural resource for roads, concrete, and other engineering applications On the other hand, Chhidru Formation aggregates should be used with extra care in concrete construction projects due to the existence of certain deleterious contents.

### Recommendation

The laboratory analysis recommended that the Late Permian Wargal Limestone, and Chhidru Formation can be used for a variety of construction projects. However, it is necessary to quantitatively confirm the presence of some detrimental contents in the Chhidru Formation, such as reactive quartz and clay, through XRD analysis and the mortar bar method of the Alkali-Silica Reaction test. Furthermore, a clearer explanation of the practical nature may be achieved by evaluating other physico-mechanical characteristics, such as the triaxial compressive strength, shear strength, young modulus, flexural modulus, shear modulus, electrical resistance, S-wave, poison's ratio, modulus rupture, and different asphalt tests.

## Data Availability

Data presented in the study are available on request from the first author and corresponding.
